# Detection of Aerosolized Protein Using a Condensation Growth Tube Coupled with an Electrochemical Immunoassay on Screen-Printed Carbon Electrodes

**DOI:** 10.1021/acs.analchem.6c00185

**Published:** 2026-04-21

**Authors:** Joowon Park, Thaisa A. Baldo, Bethany Unruh, Braden Stump, Dominick Heskett, Pat Keady, Brian Annis, Brian J. Geiss, David S. Dandy, Charles S. Henry

**Affiliations:** † Department of Chemistry, 3447 Colorado State University, Fort Collins, Colorado 80523, United States; ‡ Aerosol Devices, a division of Handix Scientific, Fort Collins, Colorado 80525, United States; § Department of Microbiology, Immunology and Pathology, 3447 Colorado State University, Fort Collins, Colorado 80523, United States; ∥ School of Biomedical and Chemical Engineering, 3447 Colorado State University, Fort Collins, Colorado 80523, United States

## Abstract

Rapid point-of-need detection of aerosolized pathogens remains a critical need for environmental monitoring and infection control. Traditional filter-based bioaerosol collection methods are time-consuming and nonselective, capturing both infectious and noninfectious viral particles while causing mechanical damage through desiccation and mechanical stress. Furthermore, these approaches require laboratory-based nucleic acid extraction and amplification, which limits their utility for real-time monitoring. Here, we present an integrated aerosol-to-detection platform that couples a three-stage condensation growth tube (CGT) bioaerosol sampler with sensitive electrochemical immunodetection of the SARS-CoV-2 nucleocapsid (N) protein as a model virus antigen. The CGT gently concentrates aerosolized particles into a small liquid volume while preserving biological integrity through a water-based, low-velocity collection. Screen-printed carbon electrodes (SPCEs) were modified with capture antibodies and employed in a sandwich immunoassay format using horseradish peroxidase-linked detection antibodies and 3,3′,5,5′-tetramethylbenzidine (TMB) as the electrochemical probe. The electrochemical immunoassay achieved a limit of detection (LOD) of 1.2 ng/mL for spiked N-protein solutions, demonstrating comparable sensitivity to published electrochemical biosensors for SARS-CoV-2 detection. The CGT successfully captured the aerosolized N protein with high efficiency across particle sizes of 50–100 nm with collection efficiency exceeding 95% for the target size range. Electrochemical detection of CGT-captured aerosolized N protein confirmed the successful combination of the sampling and sensing platform, providing average cathodic current signals distinguishable from blank controls. These results demonstrate the feasibility of coupling efficient bioaerosol collection to rapid electrochemical detection for point-of-need pathogen monitoring.

## Graphical abstract

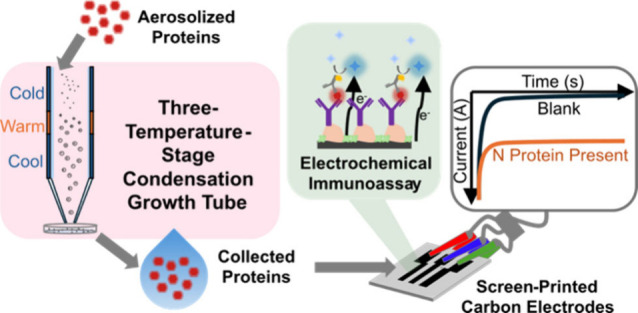

## Introduction

Aerosol sampling gained significant importance during the COVID-19 pandemic due to the potential role of airborne virus transmission in disease spread. Moreover, studying aerosol samples is essential for understanding virus dissemination dynamics, evaluating infection risk across various environments, and implementing effective preventive measures to mitigate the spread of contagious diseases.^1,2^ However, very few systems can sample bioaerosols and detect them in near real time. Specifically, current methods for studying aerosolized viruses primarily rely on filters to capture viral particles, which are then analyzed in a laboratory setting.^3,4^ These tests can provide insight into the viral load in the environment of interest, but they are limited because the filters capture both infectious and noninfectious viral particles.^5–7^ Filters can also adversely affect the viral particle integrity due to mechanical stress and desiccation, making it difficult to determine the infective fraction of the collected viruses.^8–10^ Further, current filter analysis methods require time-consuming nucleic acid extraction and amplification, which are not suitable for rapid, point-of-need monitoring.^6,11,12^ Therefore, there is a need for instruments capable of detecting aerosolized pathogens at the point-of-need to enable near-real-time analysis of environmental pathogens and support efficient disease control.^13^

There has been growing interest in developing user-friendly and cost-effective sensors for detecting pathogenic proteins from infectious viruses such as COVID-19.^14^ Electrochemical biosensors have emerged as a leading technology for this purpose, offering high sensitivity, fast response times, and seamless integration with portable electronics such as smartphones.^15^ Among these, sensors built on commercial screen-printed carbon electrodes (SPCEs) provide a practical platform, combining low reagent consumption and ease of functionalization with various biorecognition elements. Their small size and adaptability make them ideal for decentralized monitoring of viral biomarkers in both clinical and environmental settings.^16,17^ In this work, SPCEs were functionalized with antibodies specific to SARS-CoV-2 nucleocapsid (N) protein, our model virus antigen, for a detection platform that could be linked with bioaerosol collection for semi real-time monitoring. Although the SARS-CoV-2 N protein is within the virion and is accessible by disrupting the structure, it was selected as our model target protein because of its high abundance and stability throughout the infection process.^18–20^

The integration of bioaerosol sampling technologies with sensitive detection platforms represents a promising approach to address persistent challenges in monitoring aerosolized pathogens.^21–23^ Condensation growth tube (CGT) technology has demonstrated remarkable efficiency in capturing viable aerosolized pathogens while preserving their viability.^24^ This gentle, water-based collection method can capture particles ranging from 5 to 10 nm to several micrometers, maintaining biological activity without the mechanical stress or desiccation associated with traditional filter-based approaches.^25^ When coupled with sensitive electrochemical immunoassays, such integrated systems offer the potential for rapid, quantitative detection of viral biomarkers directly from environmental or clinical aerosols.^19,26^ Here, we present the coupling of an efficient bioaerosol sampling technology with a sensitive electrochemical immunosensor for detecting the SARS-CoV-2 nucleocapsid protein in both spiked solutions and aerosolized samples. This work demonstrates the feasibility of aerosol-based detection systems that could facilitate rapid, noninvasive, sensitive, and practical monitoring of viral bioaerosols.

## Materials and Methods

### Materials and Reagents

Protein A, sodium hydroxide (≥98%), sodium chloride (≥99%), sodium tetraborate (≥99%), Tween 20, Tween 80, 3,3′,5,5′-tetramethylbenzidine (TMB), and casein from bovine milk (87–94%) were purchased from Sigma-Aldrich (Saint Louis, MO). Boric acid (≥99.5%) and Pierce stable peroxide buffer (10×) was obtained from Thermo Fisher Scientific (Waltham, MA). SARS-CoV-2 (2019-nCoV) nucleocapsid-His recombinant protein, SARS-CoV/SARS-CoV-2 nucleocapsid antibody, and horseradish peroxidase (HRP)-linked SARS-CoV/SARS-CoV-2 nucleocapsid antibody were acquired from Sino Biological (Beijing, China). Igepal CA-630 was purchased from MP Biomedicals (Santa Ana, CA). Phosphate buffer saline (PBS) tablets were obtained from Medicago (Quebec, Canada). Polyethylene terephthalate (PET) transparency film (9984) and double-sided adhesive (DSA) sheets (468) were purchased from 3 M (Saint Paul, MN). Carbon ink (CI-2057) was acquired from Engineered Materials Solutions (Attleboro, MA). Silver conductive paint was purchased from Structure Probe, Inc. (West Chester, PA). Granular ammonium sulfate (≥95%) was obtained from Lab Alley (Austin, TX). Preparations of solutions are specified in the Supporting Information.

### Modification of Screen-Printed Carbon Electrodes

Commercial screen-printed carbon electrodes (SPCEs, DropSens DRP-110-U75) were modified with SARS-CoV/SARS-CoV-2 nucleocapsid antibody (capture antibody), which acts as the biorecognition element for SARS-CoV-2 nucleocapsid-His recombinant protein (N protein). 5 μL of 5 μg/mL Protein A was passively adsorbed on the defined working electrode (WE) and dried at 37 °C for 20 min. Then, 5 μL of 10 μg/mL capture antibody was added onto the WE, incubated in a humidity chamber for 1 h, and washed with 10 mM PBS. Next, the WE was incubated with 5 μL of 0.3% aged casein for 1 h in a humidity chamber to block nonspecific binding sites and washed with 10 mM PBS.^27^

### Preparation and Analysis of Aerosol Sample

Aerosol Devices Inc. has developed a condensation growth tube (CGT) collector, the BioSpot-VIVAS bioaerosol sampler, which has been rigorously documented^28^ to be effective at capturing aerosolized virus. However, for this experiment and later field applications, the size, weight, and power draw of the instrument were too great to easily fit into fume hoods or for transport to future test sites. To better meet the needs of the project, a new form factor of the CGT was designed, named the BioSpot-VITA bioaerosol sampler. The design of this new instrument followed the standard structure of a CGT as previously designed in the VIVAS, but it was reduced in volume by 33%, reduced in weight by 40%, and reduced in power draw by 30% (Figure S1).

#### Size-Selected Collection Efficiency

The new form factor of the CGT sampler designed for this project required confirmation testing of its ability to capture airborne particles in the size range of 5 nm to 10 μm. The experimental setup for size-selected collection efficiency testing is shown in Figure S2. Ammonium sulfate challenge aerosol was generated using a nebulizer (Topas ATM 210) and diffusion dryer. The concentration of the challenge aerosol was controlled using a dilution bridge feeding HEPA-filtered air into the aerosol stream. A differential mobility analyzer (TSI DMA 3081) strips out a narrow size fraction of the aerosol using electrical mobility classification. The concentration of the challenge aerosol upstream and downstream of the sampler was measured with a versatile water condensation particle counter (CPC, TSI water-CPC 3789).

The collection efficiency of the CGT instrument to capture particles of a certain size was calculated by the relationship between the aerosol concentration upstream of the CGT (*C*_Upstream_) and the aerosol concentration downstream of the CGT (*C*_Downstream_) by the following equation:

$$Collection\;Efficiency\;\left[\%\right]=\frac{C_{Upstream}-C_{Downstream}}{C_{Upstream}}\times 100$$ 1

The above efficiency calculation was completed at 17 different particle sizes using the DMA between 6 and 200 nm, as those sizes are where the CGT is expected to greatly increase the capture efficiency of the particles. Above 200 nm, it is expected that aerosol will be captured with the same or greater efficiency as 200 nm particles, as they will be easier to condense onto and inertially capture, and below 6 nm is outside of the scope of the design of this CGT.^29^

#### N Protein Particle Size and Distribution

After completing collection efficiency testing to confirm the expected operation of the CGT, the N protein solution was aerosolized and analyzed for the capture and subsequent sensing using an electrochemical immunoassay. A solution of 2.5 mL of buffer containing 83 ng/mL N protein or a control buffer without added protein was placed into the nebulizer (Topas ATM 210) to generate an N-protein-laden aerosol. An additional 2.5 mL of buffer was added to the nebulizer cup to meet the minimum required liquid volume in the nebulizer, resulting in a total liquid volume of 5 mL. The 15 min aerosol sample and particle size distribution were recorded, and then, the 90 min aerosol sample and particle size distribution were recorded directly after that (Figure 2). The concentration of the test aerosol was controlled by using a dilution bridge feeding HEPA-filtered air into the aerosol stream. Using a differential mobility analyzer (TSI DMA 3081) and a versatile water condensation particle counter (CPC, TSI water-CPC 3789) in concert creates a scanning mobility particle sizer (SMPS), which was used to measure the particle size distribution of the N-protein-laden aerosol output downstream of the dilution bridge. This distribution is useful for determining the particle-size distribution entering the CGT for capture and subsequent off-line analysis.

### Electrochemical Immunoassay

#### Spiked N Protein in Solution

Sample solutions containing desired concentrations (0.5, 1, 2, 5, 10, 50, 100, 500, 1000, 2000 ng/mL) of N protein were prepared by spiking in SARS-CoV-2 (2019-nCoV) nucleocapsid-His recombinant protein in 1× stable peroxide buffer (SPB). Three μL of sample solution of 1× SPB for blank measurements was added to the defined WE and incubated in a humidity chamber for 30 min. The electrodes were washed with PBST (10 mM PBS, 0.05% Tween 20), followed by 10 mM PBS using spray bottles. Next, 3 μL of 2.5 μg/mL HRP-linked SARS-CoV/SARS-CoV-2 nucleocapsid detection antibody in 1× SPB was pipetted onto the WE and incubated for 25 min in a humidity chamber. Electrodes were washed with PBST and PBS spray bottles. Subsequently, 50 μL of a commercially available, ready-to-use TMB solution was added to the electrode surface, covering all three electrodes (working, counter, and reference electrode), and incubated for 2 min. Right after the incubation, a portable potentiostat (EmStat4s-PalmSens) was used to apply a fixed potential to the WE (0.0 V vs Ag) for 2 min.

#### Aerosolized and Captured N Protein Samples

Every step of the experiments to quantify N protein in aerosolized and captured samples was identical with those for quantifying spiked N protein in solution, except for the sample solution preparation step. Instead of spiking the desired amount of target protein into the buffer, 83 ng/mL N protein in 10 mM PBS was aerosolized and recaptured using the CGT as described in the section N Protein Particle Size and Distribution. The resulting liquid sample was used as the sample solution.

#### Electrochemical Data Analysis

Reduction current signals were collected by averaging the current in chronoamperometry measurements at 55–65 s time points, where the chronoamperograms plateaued. All electrochemical measurements were made in triplicate using three separately modified SPCEs, and the average values were plotted along with error bars indicating standard deviations.

## Results and Discussion

### Analysis of Aerosol and Particles

The collection of aerosolized samples utilized the CGT capture technology. The core sampling technology is a three-stage laminar-flow, water-based condensational growth tube^28,30–33^ (Figures 1 and S1) that collects and concentrates bioaerosol particle sizes from 10 to 10,000 nm into a small volume of liquid (∼1.5 mL), making it more effective at sampling naked virus particles such as SARS-CoV-2 (∼120 nm) as well as viruses encased in droplet secretions in the 0.3 to 10 μm size range. The 8 LPM sample flow rate approximates the breathing rate of an adult and is achieved by employing six parallel growth tubes with 1.33 LPM flowing through each tube.^28,32^ The temperatures and humidity of the CGT mimic the environment in the human lung. Particles enter the CGT, whose internal wall is lined with a thin wick that is continuously saturated with liquid water. As aerosol moves through the cold (5 °C) conditioner, it becomes saturated with water vapor, and a baseline temperature is established independent of ambient conditions. Supersaturation occurs downstream of the conditioner in the initiator due to the difference in the diffusivity of water vapor and thermal energy. The warm (45 °C) walls of the initiator heat the sample air and increase the partial pressure of water vapor. Since water vapor diffuses more rapidly in air than thermal energy, the water vapor diffuses into the flow faster than the flow warms. Thermodynamic equilibrium drives the supersaturated air to condense water vapor on the seed particles. The high supersaturation in the initiator (∼140%) activates condensational growth of particles as small as 5–10 nm in diameter. Once activated, the particles grow through condensation as they pass through the final moderator section (10–20 °C) to form >4 μm sized droplets that are readily collected with gentle, low-velocity impingement into a liquid (water, buffer, or nutrient broth). The aerosol-droplet stream is split into multiple jets, lowering the velocity in each jet for gentle dimpling at the air–liquid interface and avoiding reaerosolization. The water encapsulating the viruses and gentle collection prevents desiccation or mechanical stresses and maintains the viability of the virus. The concentrated particle suspension in the sample increases detection sensitivity.

**Figure 1:**
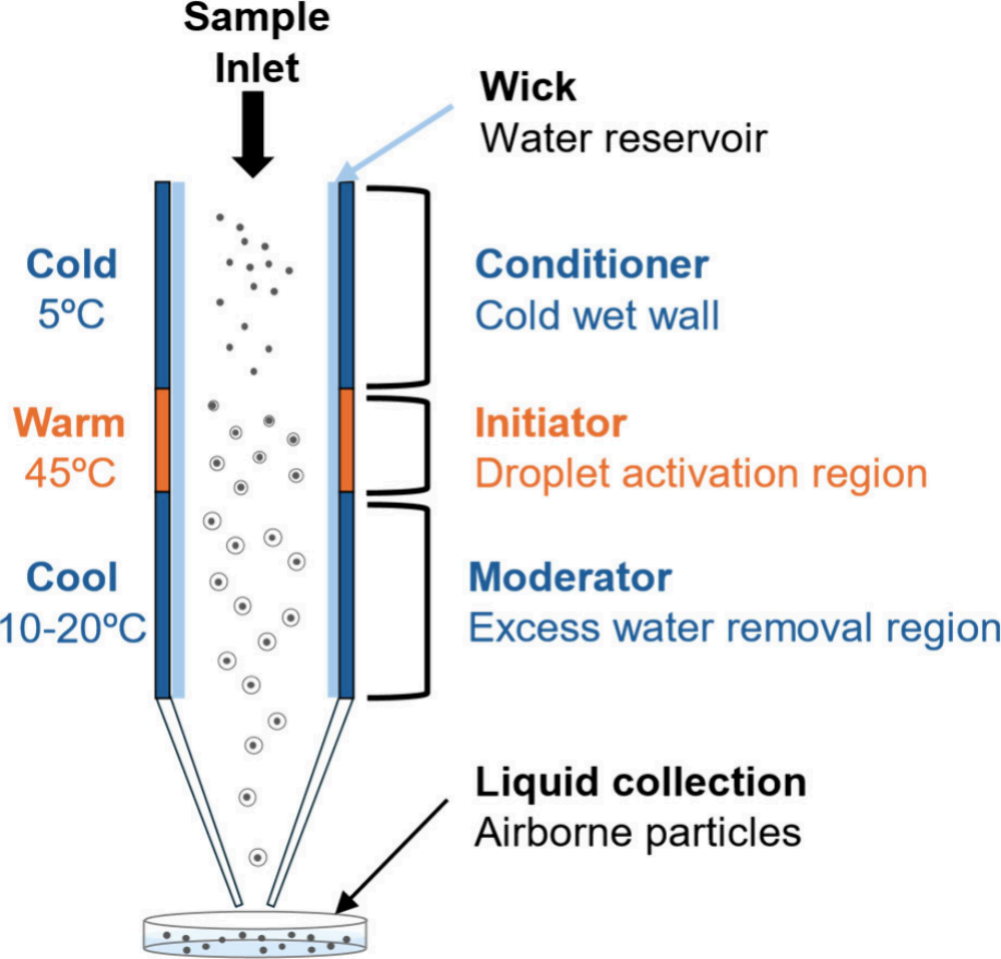
Schematic drawing of the three-temperature-stage condensation growth tube (CGT) for capturing bioaerosols into liquid form.

#### Collection Efficiency Analysis

Particle size can greatly affect the efficiency of aerosol collection. Without the utilization of a CGT, it would be very difficult to inertially capture particles 6–200 nm without damaging them due to high mechanical stresses or desiccation from high velocity air flow.^34,35^ The CGT condensing water around particles into larger droplets approximately 4 μm in size allows for gentle, lower-velocity inertial impaction to occur. Figure S3 shows that less than 5% of ammonium sulfate particles counted as entering the instrument were counted downstream using both the commercially available BioSpot-VIVAS and the scaled-down BioSpot-VITA primarily used in this article, implying the efficient collection of particles in a wide size range.

#### Aerosolization and Capture of N Protein

The aerosolization and capture of N protein using the BioSpot-VITA CGT were performed and analyzed. The size distribution of the aerosolized N protein is displayed for the two experiments performed (CGT sampling for 15 and 90 min, respectively) in Figure 2. For 15 min sampling, the total particle density was 37,700 counts/cm^3^ and the geometric mean size of the particles was 39.2 nm with a standard deviation of 2.3. For 90 min, the total particle density was 98,000 counts/cm^3^ and the geometric mean size of the particles was 62.2 nm with a standard deviation of 2.1. These distributions imply that the largest number of particles entering the CGT from the aerosolized N protein are 50–100 nm in size, which is within the range that the CGT should have a collection efficiency higher than 95%, as shown in Figure S3. It is also important to note that the 90 min sampling had approximately 2.6 times higher total aerosol density than the size distribution of 15 min sampling. This change in aerosol density may be related to several settings requiring greater control or monitoring of the nebulizer, the dilution bridge, and the CGT. Without proper control on the amount of aerosol entering the instrument, it is difficult to quantify the effect that longer sampling time has on improved assay performance.

**Figure 2:**
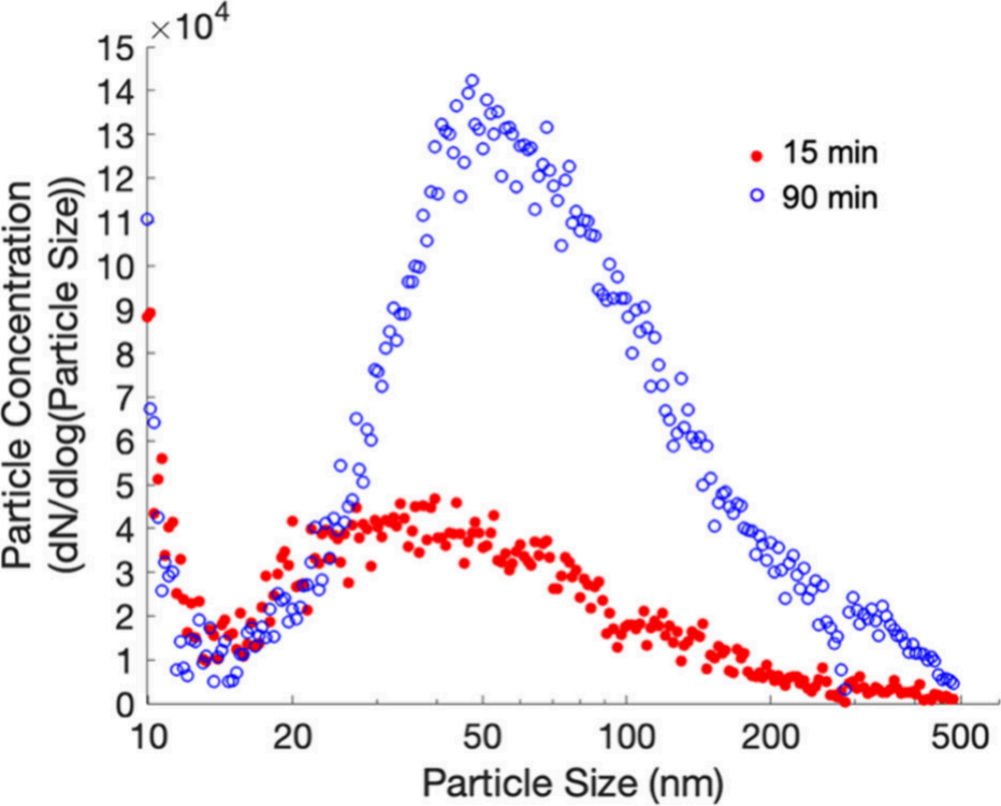
Particle size distribution of N protein-laden aerosol was analyzed using a scanning mobility particle sizer (SMPS).

### Electrochemical Detection of SARS-CoV-2 N Protein

An electrochemical sandwich immunoassay was used to detect SARS-CoV-2 N protein. Screen-printed electrodes were fabricated to form a three-electrode system with a carbon working electrode, a carbon counter electrode, and a silver pseudoreference electrode (RE). The working electrode was subsequently modified with protein A, SARS-CoV/SARS-CoV-2 nucleocapsid capture antibody, and 0.3% aged casein, which served as a blocker inhibiting nonspecific binding of nontarget molecules as shown in Figure S4. Here, protein A was passively adsorbed to aid with orienting the capture antibody so that it could be more readily available for N protein to bind.^36–38^ Subsequently, the modified SPCEs were incubated with liquid sample that contains N protein so that binding between capture antibody and N protein would occur. The N protein sample was prepared either by spiking recombinant SARS-CoV-2 N protein in 1× SPB or by the aerosolization and capture process using a three-stage laminar-flow, water-based CGT (BioSpot-VITA). Next, HRP-linked SARS-CoV/SARS-CoV-2 nucleocapsid detection antibody (detection antibody) was pipetted onto the WE for it to bind to N protein. 3,3′,5,5′-Tetramethylbenzidine (TMB) acted as the electrochemical probe in this system. HRP linked to the detection antibody oxidized TMB to form a charge transfer complex that could be reduced by the potential applied to the working electrode (0.0 V vs Ag). Consequently, increased reduction current signal was registered when N protein was present in the sample compared to the blank. The cathodic current was represented by the average current in chronoamperometry measurements at 55–65 s time points. In this work, the electrochemical immunoassay was used to confirm the ability to couple CGT-based bioaerosol sampling and electrochemical detection.

Validation of the experimental parameters, such as concentrations and incubation conditions of protein A, capture antibody, and blockers, was not investigated in depth because extensive optimization of a similar biomolecular setup on SPCEs for SARS-CoV-2 N protein sandwich immunoassays has already been reported.^19,39^ Electrochemical and morphological characterization of the step-by-step modified electrodes has also been described previously using cyclic voltammetry, scanning electron microscopy, and profilometry.^19^ Therefore, experimental conditions previously demonstrated to be effective were adopted here without modification to focus on evaluating the successful combination of bioaerosol collection with electrochemical detection. Future work may involve targeted fine-tuning of assay parameters for CGT-collected aerosolized proteins to improve analytical performance.

#### Spiked N Protein in Solution

The analytical performance of the electrochemical immunoassay was first assessed by using 1× SPB spiked with known amounts of recombinant N protein. Figure S5 shows representative chronoamperograms and a dose–response curve generated by analyzing samples with multiple N protein concentrations (0, 0.5, 1, 2, 5, 10, 50, 100, 500, 1000, 2000 ng/mL). The plateau TMB reduction signal increased with the concentration of N protein in solution. The dose–response curve was fitted using a 4-parameter logistic (4PL) curve, and the limit of detection (LOD) was calculated as the theoretical blank plus three times the standard deviation of the blank. The LOD of the electrochemical immunoassay for spiked N protein solution was calculated to be 1.2 ng/mL, comparable to other published electrochemical biosensors^40,41^ for SARS-CoV-2 N protein detection.

#### Aerosolized and Captured N Protein Samples

To demonstrate the ability to capture N proteins in aerosol form into a liquid sample and to detect them, 83 ng/mL N protein in PBS was nebulized and recaptured using the water-based, laminar-flow CGT system. Figure 3 shows representative chronoamperograms and average plateau cathodic current signals for electrochemical immunoassays performed for samples prepared either directly in solution on a laboratory bench or by using CGT. For samples prepared directly in solution, the blank and 83 ng/mL signals from 1× SPB were larger compared to the corresponding control and 83 ng/mL samples prepared by collecting aerosol for 15 and 90 min for liquid form collection using CGT. This discrepancy is likely due to the loss of nonwater components such as sodium chloride, surfactants, peroxides, and N protein in the case of positive samples during the nebulization and condensation process. The concentration of 83 ng/mL was chosen as a compromise between maintaining a measurable electrochemical signal and preserving sufficient sample volume for aerosol collection. Preliminary tests using 100 ng/mL produced detectable responses but would have consumed an excessive portion of the available protein stock, leaving insufficient volume for additional studies. When comparing samples prepared by collecting aerosol for 15 and 90 min for CGT input, the overall cathodic current signal was smaller for the 90 min collection. Two viable explanations exist: protein unfolding or decline in CGT performance. First, N proteins captured in liquid with low ionic strength are subject to unfolding, which directly results in impaired recognition by the capture and detection antibodies in the immunoassay. Second, although it might be expected that longer collection time would yield greater aerosol capture and thus higher signals, it has been reported^42^ that, depending on design, CGT instruments experience reductions in droplet growth performance due to higher particle density. The smaller electrochemical TMB reduction signal using the sample prepared by 90 min sampling, where aerosol density was much higher compared to 15 min sampling, suggests improvement to the design of the CGT to be able to operate in a variety of particle density conditions. Specifically, decreasing the length of the conditioner stage in the CGT in favor of increasing the length of the initiator and moderator stages allow for droplet growth to occur for a longer amount of residence time for each particle, better guaranteeing particles are condensed enough to be captured with high efficiency even when in high density conditions. It is also important to note that the suggested high density of particles in 90 min sampling (98,000 counts/cm^3^) is unlikely to occur in real-life settings, such as during a human or animal challenge study or in ambient settings.

**Figure 3:**
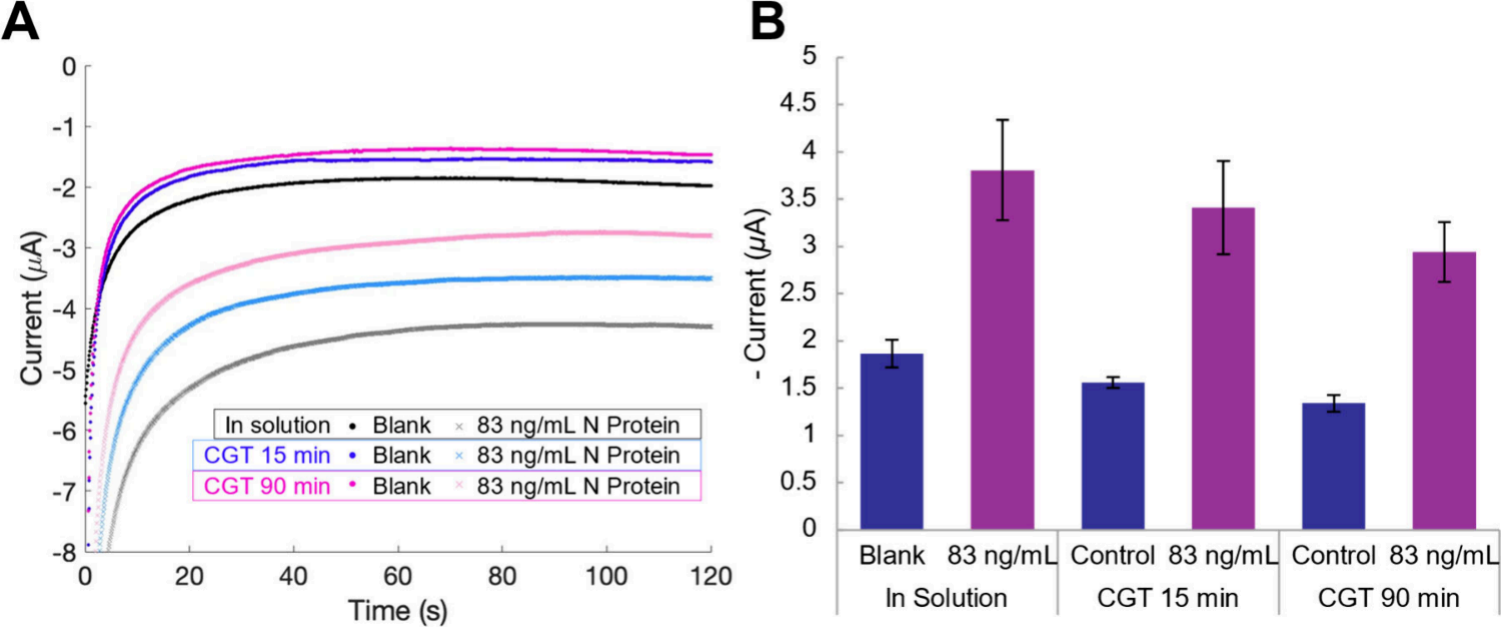
Chronoamperometric measurements showing the performance of the electrochemical immunoassay for the detection of SARS-CoV-2 nucleocapsid (N) protein spiked in solution or in samples collected using the CGT for 15 and 90 min. (A) Representative chronoamperograms and (B) average current signal of the plateau region (55–65 s). Error bars indicate standard deviations of the average currents (*n* = 3).

## Conclusion

This work demonstrates the successful combination of efficient bioaerosol sampling technology with a sensitive electrochemical immunoassay, establishing a platform for rapid bioaerosol monitoring. The coupling of the BioSpot-VITA system with screen-printed electrode assays enables sensitive detection of the SARS-CoV-2 nucleocapsid protein as a model pathogen in aerosolized samples, directly addressing the limitations of traditional laboratory-based methods that require time-consuming nucleic acid extraction and amplification. The platform offers substantial practical advantages for point-of-need deployment. The redesigned BioSpot-VITA bioaerosol sampler provides significant improvements in portability and operational efficiency compared to its predecessor, enabling deployment in diverse field settings, including fume hoods and challenging environments. The gentle collection mechanism preserves bioaerosol integrity while concentrating particles into a liquid suspension, substantially increasing detection sensitivity compared to dry collection approaches.

Future improvements can take several forms. Using pathogens such as intact viruses, intact bacteria, or surface proteins rather than the N protein will better reflect real-world conditions. Similarly, replacing the nebulized sample solution with human breath samples will also more closely mimic practical use. Regarding the sampling instrument, a deeper investigation into the effects of operational parameters across each component of BioSpot-VITA (nebulizer, dilution bridge, CGT) on the total collected particle density will help improve the understanding and performance of bioaerosol sampling. In addition, depositing the aerosol sample onto the working electrode rather than a Petri dish would simplify collection, accelerate analysis, and reduce sample loss, since the CGT only requires a surface a few millimeters below its nozzles for efficient capture. Specifically, placing a three electrode system with a circular working electrode beneath the exit nozzle of the CGT would allow immediate off-line immunoassay with improved recovery. Ultimately, incorporating microfluidic devices will enable automated immunoassays, minimizing sample handling and user steps. In conclusion, this work serves as the basis for advances to enable rapid, noninvasive, sensitive, and practical aerosol-based biosensing.

## Supplementary Material



## Data Availability

Data will be made available on request.

## Notes

Method S1: preparation of solutions; illustration and photo of CGT sampler; flowchart of the size-selected particle collection efficiency test setup; collection efficiency with various particle sizes; schematic illustration of the electrochemical immunoassay; representative chronoamperograms of electrochemical immunoassays and dose-response scatter plot (PDF)
