# Supplementary material for: Detection of Aerosolized Protein Using a Condensation Growth Tube Coupled with an Electrochemical Immunoassay on Screen-Printed Carbon Electrodes
Source: Anal Chem. 2026 Apr 21;98(17):12201–7. doi: 10.1021/acs.analchem.6c00185 (PMC13150808; doi:10.1021/acs.analchem.6c00185)
Supplement: Supplementary file 1 [file ac6c00185_si_001.pdf]

Supporting Information

Detection of Aerosolized Protein Using a Condensation Growth Tube Coupled with an Electrochemical Immunoassay on Screen-Printed Carbon Electrodes

Joowon Park[a+], Thaisa A. Baldo[a+], Bethany Unruh[a], Braden Stump[b], Dominick Heskett[b], Pat Keady[b], Brian Annis[b], Brian J. Geiss[c.d.], David S. Dandy[d], and Charles S. Henry[a.d]*

[a] Department of Chemistry, Colorado State University, CO, USA, 80523

[b] Aerosol Devices, a division of Handix Scientific, CO, USA, 80525

[c] Department of Microbiology, Immunology and Pathology, Colorado State University, CO, USA, 80523

[d] School of Biomedical and Chemical Engineering, Colorado State University, CO, USA, 80523

[+]These authors contributed equally.

[*]Corresponding author. chuck.henry@colostate.edu

Table of Contents

	Topic	Page
**Method S1**	Preparation of solutions	S3
**Fig. S1**	**(A)** Illustration of the interior design of the CGT sampler BioSpot-VITA™, and **(B)** a photograph of the assembled CGT prototype.	S4
**Fig. S2**	Flowchart of the size-selected particle collection efficiency test setup. Two condensation particle counters (CPC, TSI 3789) are used to measure the concentration of size-selected particles upstream and downstream of the CGT sampler.	S5
**Fig. S3**	Collection efficiency with various particle sizes was tested using two condensation particle counters (CPC), each measuring upstream and downstream of the three-temperature-stage CGT sampler.	S6
**Fig. S4**	Schematic illustration of the electrochemical immunoassay employed to quantitatively detect SARS-CoV-2 N protein.	S7
**Fig. S5**	**(A)** Representative chronoamperograms of electrochemical immunoassays detecting different N protein concentrations (0, 0.5, 1, 2, 5, 10, 50, 100, 500, 1000, 2000 ng/mL) in solution. **(B)** A dose-response scatter plot was built using the average plateau currents between 55 and 65 sec of each chronoamperogram. The scatter plot was fitted using a 4-parameter logistic (4PL) curve. Error bars indicate standard deviations of the average currents (n=3).	S8
**References**		S9

Method S1. Preparation of solutions

All solutions were made using purified water prepared through the Milli-Q system (18.2 MΩ) from Sigma-Aldrich (Saint Louis, MO). The 6% aged casein[1] stock was prepared by dissolving 6 g of casein from bovine milk in 80 mL of 50 mM sodium hydroxide solution overnight and adding 0.26 g of boric acid and 0.45 g of sodium tetraborate, followed by adjusting the pH to 8.5 and the volume to 100 mL with purified water the next day. The prepared 6% casein solution was incubated at 37 °C for 7 days to result in 6% aged casein stock solution. 6% aged casein stock was aliquoted into 50 mL microcentrifuge tubes, stored at -20 °C to be thawed right before use, and diluted with 50 mM borate buffer (pH 8.5) to the desired concentration. 10 mM PBS (pH 7.4) was prepared by dissolving one tablet in 500 mL of purified water. PBST was prepared similarly, with additional content of 0.05% Tween® 20. 1× stable peroxide buffer (SPB) was prepared by diluting Pierce™ stable peroxide buffer (10×) by 10-fold, and by adding 300 mM sodium chloride, 0.1% Tween® 80, and 0.1% Igepal.

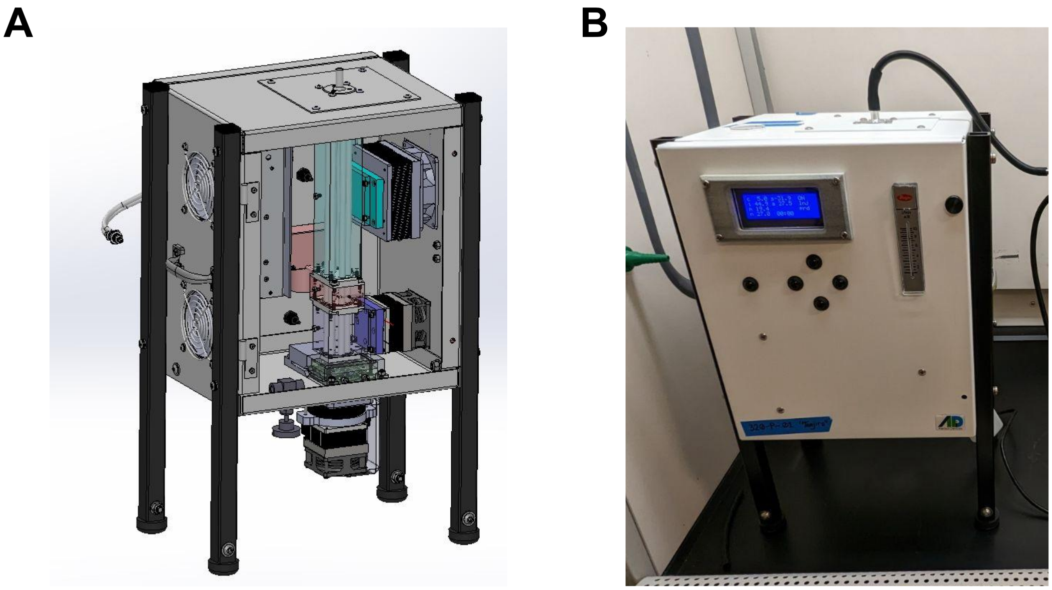

Figure S1. (A) Illustration of the interior design of the CGT sampler BioSpot-VITA™, and **(B)** a photograph of the assembled CGT prototype.

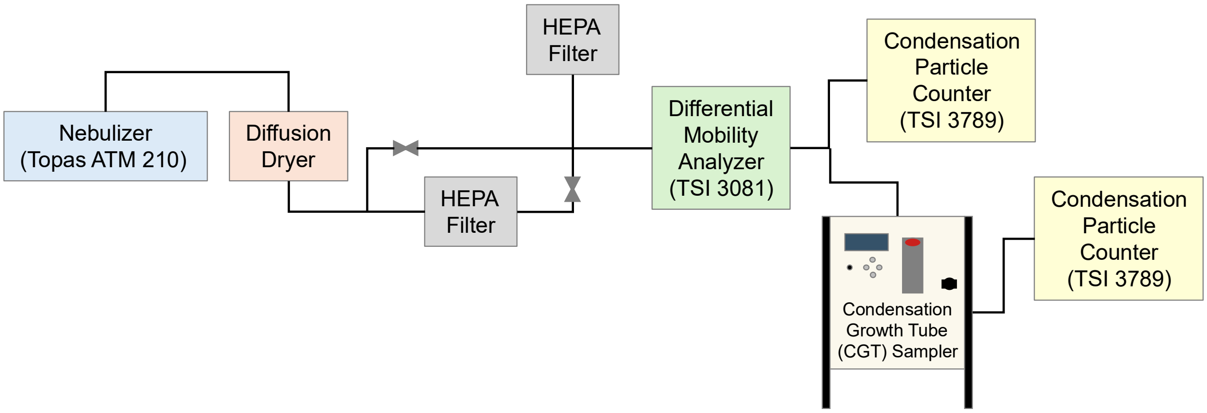

Figure S2. Flowchart of the size-selected particle collection efficiency test setup. Two condensation particle counters (CPC, TSI 3789) are used to measure the concentration of size-selected particles upstream and downstream of the CGT sampler.

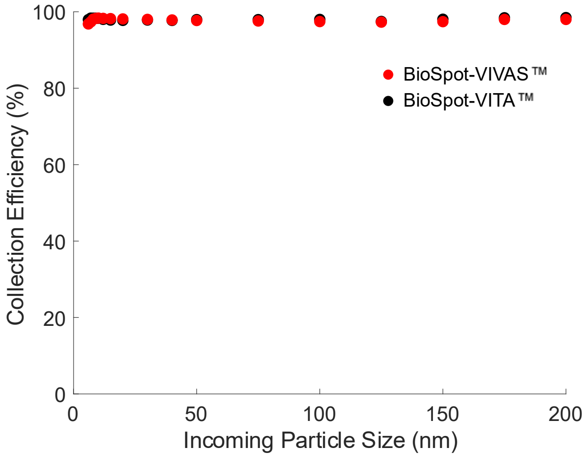

Figure S3. Collection efficiency with various particle sizes was tested using two condensation particle counters (CPC), each measuring upstream and downstream of the three-temperature-stage CGT sampler.

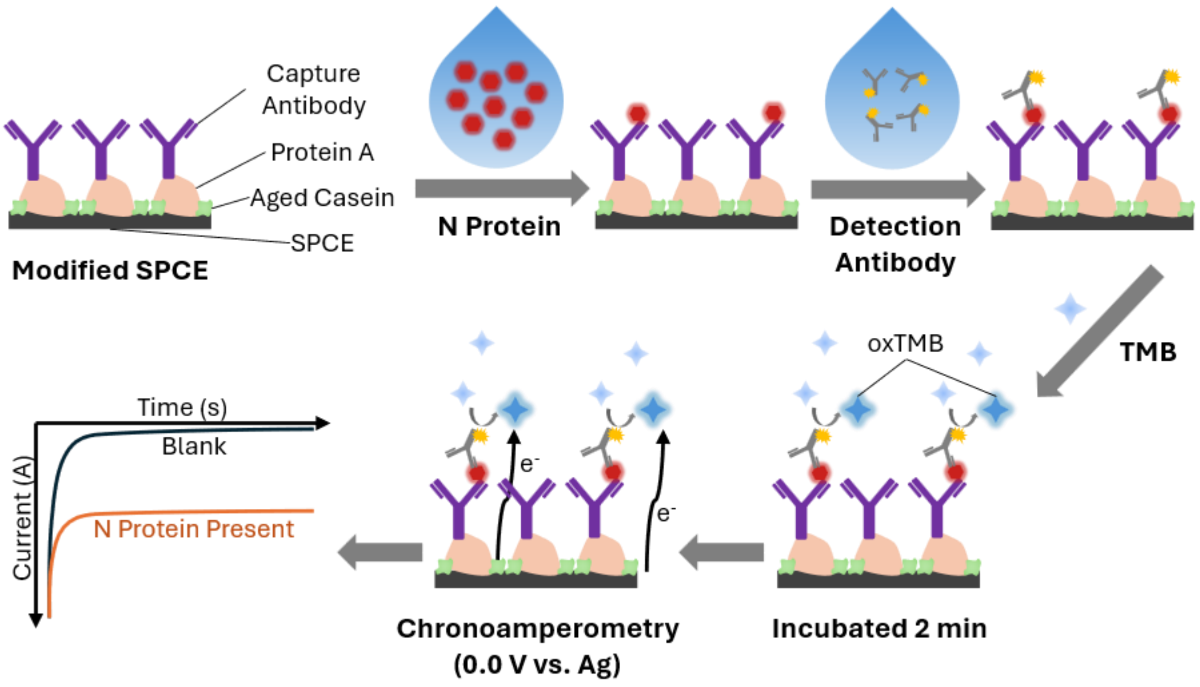

Figure S4. Schematic illustration of the electrochemical immunoassay employed to quantitatively detect SARS-CoV-2 N protein.

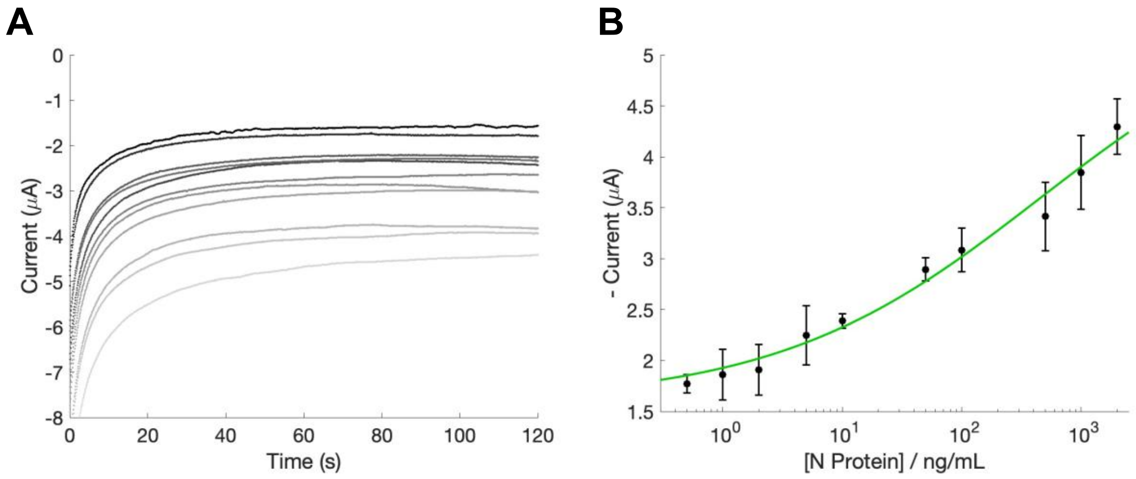

Figure S5. (A) Representative chronoamperograms of electrochemical immunoassays detecting different N protein concentrations (0, 0.5, 1, 2, 5, 10, 50, 100, 500, 1000, 2000 ng/mL) in solution. **(B)** A dose-response scatter plot was built using the average plateau currents between 55 and 65 sec of each chronoamperogram. The scatter plot was fitted using a 4-parameter logistic (4PL) curve. Error bars indicate standard deviations of the average currents (n=3).

References

(1) Grant, B. D.; Anderson, C. E.; Williford, J. R.; Alonzo, L. F.; Glukhova, V. A.; Boyle, D. S.; Weigl, B. H.; Nichols, K. P. SARS-CoV-2 Coronavirus Nucleocapsid Antigen-Detecting Half-Strip Lateral Flow Assay Toward the Development of Point of Care Tests Using Commercially Available Reagents. *Anal. Chem.* **2020**, *92* (16), 11305–11309. https://doi.org/10.1021/acs.analchem.0c01975.
